# Microwave Ablation for the Treatment of Non-Colorectal Cancer Liver Metastasis

**DOI:** 10.3390/cancers18132026

**Published:** 2026-06-23

**Authors:** Jacopo Lanari, Sara Lazzari, Ilaria Billato, Chiara Naldini, Clarissa De Nardi, Giulia Tamponi, Davide Volpato, Alessandro Furlanetto, Francesco Enrico D’Amico, Alessandro Vitale, Enrico Gringeri, Umberto Cillo

**Affiliations:** 1 Department of Surgical, Oncological and Gastroenterological Sciences, University of Padua, 35128 Padua, Italy; 2 General Surgery and Hepato-Pancreato-Biliary Surgery and Liver Transplantation Unit, Padua University Hospital, 35128 Padua, Italy; 3 Department of Biology (DiBio), University of Padua, 35128 Padua, Italy

**Keywords:** microwave ablation, thermal ablation, liver metastasis, survival, textbook outcome

## Abstract

**Background**: Non-colorectal cancer liver metastases (NCRLMs) represent a therapeutically challenging condition with poorly defined locoregional treatment options. This study evaluates the safety, oncological efficacy, and survival outcomes of microwave ablation (MWA) for NCRLMs in a large single-centre series. **Methods**: Retrospective analysis of patients undergoing MWA for NCRLM between January 2010 and December 2024 at a high-volume hepatobiliary centre. Endpoints were safety, efficacy, textbook outcome (TO) achievement, and overall survival (OS). **Results**: A total of 138 patients underwent 172 MWA procedures across eight primary tumour categories via a video-assisted (*n* = 110, 64%) or percutaneous (*n* = 62, 36%) approach. Major complications (Clavien–Dindo ≥ 3) occurred in 1.8% of procedures, with a median hospital stay of 2 days and a 90-day mortality of 1.7%. The median follow-up was 24.9 (10.3, 55.8) months. Complete response (CR) was achieved in 77% of procedures and TO in 54%, with incomplete response as the main driver of TO failure. The 5-year OS varied by histology, from 100% (GIST) and 80.0% (GEP-NET) to 44.7% (breast) and 0% (pancreatic adenocarcinoma). The video-assisted ablation was associated with superior OS compared to the percutaneous one (5-year OS 54.4% vs. 26.0%, *p* = 0.00025). In the multivariable analysis, the percutaneous approach (HR 2.44), ECOG PS ≥ 2 (HR 6.06) and a higher tumour burden score (HR 1.09) independently predicted worse OS, whereas the histological group was not independently associated with OS. Repeat MWA was the most frequent treatment for first hepatic recurrence, and 50% of patients reached no evidence of disease (NED) at last follow-up. **Conclusions**: MWA is a safe and repeatable locoregional treatment for NCRLMs, with outcomes shaped by tumour biology, disease burden, patient fitness and procedural approach. The high rate of liver-dominant recurrence treated with repeat MWA, combined with a final NED rate of 50%, supports MWA as a platform for iterative locoregional disease control in selected patients.

## Simple Summary

Liver metastases from cancers other than colorectal cancer represent a growing therapeutic challenge, as many patients are not eligible for surgery and treatment options remain poorly defined. Microwave ablation is a minimally invasive technique that destroys tumour tissue using heat and can be performed through a small incision (video-assisted approach) or through the skin (percutaneous approach). In this study, we report our 14-year experience with microwave ablation in 138 patients with liver metastases from eight different cancer types, including breast cancer, neuroendocrine tumours, gastrointestinal stromal tumours, and pancreatic cancer. The procedure was safe, with few major complications and short hospital stays. Survival varied considerably depending on the type of primary cancer, and the video-assisted approach was associated with better survival than the percutaneous one. Importantly, when liver metastases recurred, microwave ablation could be repeated, allowing about half of the patients to achieve no evidence of disease at the end of follow-up. These findings support microwave ablation as a versatile and repeatable tool in the multidisciplinary management of liver metastases from non-colorectal cancers.

## 1. Introduction

Liver metastases from non-colorectal cancer represent a growing therapeutic challenge, often associated with a poor prognosis and limited treatment options. Whereas surgical resection remains the gold standard for isolated lesions, many patients are deemed inoperable due to lesion number, location, comorbidities, or underlying hepatic dysfunction. Microwave ablation (MWA) has emerged as a minimally invasive modality capable of rapidly delivering high temperatures and producing predictable, confluent zones of tumour necrosis—even in proximity to large vessels where heat-sink effects compromise alternative thermal techniques. Although MWA is well established for hepatocellular carcinoma (HCC) and colorectal cancer liver metastasis (CRLM) [1,2,3], its role in non-colorectal malignancies has been less rigorously defined [4,5]. In this retrospective, single-centre observational study, we aimed to evaluate the safety and oncological outcomes of MWA for liver metastases from non-colorectal primaries, assessed through perioperative morbidity and mortality, efficacy, textbook outcome (TO) achievement, and overall survival (OS). As secondary, exploratory objectives, we examined the influence of the procedural approach (video-assisted versus percutaneous) and of primary tumour histology on these outcomes.

## 2. Materials and Methods

This was a retrospective analysis of patients treated with MWA between January 2010 and December 2024 at General Surgery 2-Hepato-Pancreato-Biliary Surgery and Liver Transplantation Unit, Padua University Hospital, Padua, Italy.

Eligible patients were adults (≥18 years) undergoing MWA for non-colorectal cancer liver metastases (NCRLMs), whether treatment-naïve or recurrent after liver resection (LR) or other therapies. Procedures performed via open surgery (laparotomy) were excluded.

The procedure involved insertion of a 14 G water-cooled coaxial antenna into the tumour under ultrasound guidance; all ablations used a 2.45 MHz microwave generator (AMICA GEN; HS Hospital Service S.p.A., Aprilia, Italy) at a median power of 40 watts (interquartile range [IQR], 40–60).

Our centre’s selection criteria for the laparoscopic approach were derived from longstanding institutional experience with minimally invasive ablation [3,6] and were applied to the present NCRLM cohort. The laparoscopic approach was adopted when percutaneous ablation was not feasible, based on technical and anatomical criteria: lesions in critical/unfavourable locations (superficial or subcapsular nodules, caudate lobe, superoposterior or subphrenic segments, lesions adjacent to the biliary tree or major hepatic vessels), proximity to adjacent organs (colon, duodenum, gallbladder, diaphragm, and heart), severe coagulopathy (PT < 40% or platelet count < 30 × 10^9^/L), untreatable ascites, and inadequate ultrasound visualisation (e.g., obesity or previous surgery). The need for a concurrent surgical procedure also favoured the video-assisted route. These criteria, shared by other groups performing laparoscopic ablation [7], are technical and anatomical and therefore independent of tumour histology and liver function; unlike in the cirrhotic HCC setting, allocation did not depend on liver-function criteria. Thoracoscopic transdiaphragmatic liver ablations were grouped with laparoscopic procedures within the video-assisted cohort, as both represent minimally invasive surgical approaches.

Liver metastases were classified according to the tumour of origin in 8 categories as follows: (1) breast cancer; (2) gastrointestinal stromal tumour (GIST); (3) lung cancer; (4) melanoma; (5) gastro-entero-pancreatic neuroendocrine tumour (GEP-NET); (6) other gastrointestinal (GI) primaries (i.e., oesophageal, gastric, Vater’s papilla, ileal, gallbladder, perihilar and distal cholangiocarcinoma [CCA]); (7) other rare histologies (i.e., tonsil carcinoma, leiomyosarcoma, epithelioid haemangioendothelioma, as well as parotid gland, laryngeal, thymic, adrenal gland, renal, endometrial, ovarian, vaginal, and prostatic cancers); (8) pancreatic adenocarcinoma. For the multivariable analyses, the eight histological categories were grouped a priori into three prognostic categories, on the basis of the established biological behaviour of liver metastases from each primary and independently of the outcomes observed in the present cohort: a favourable category (breast cancer, GIST, and GEP-NET); an unfavourable category (lung cancer, melanoma, other GI primaries, and pancreatic adenocarcinoma); and an “other” category, comprising the heterogeneous rare histologies that could not be assigned a coherent prognostic profile a priori. This grouping allowed for adjustments for primary tumour biology while limiting the number of model parameters and avoiding the unstable estimates expected from the smallest individual subgroups.

Tumour burden (number and dimension of nodules) was measured at the last CT scan or MRI before ablation. In addition, the tumour burden score (TBS), defined as the distance from the origin on a Cartesian plane combining the maximum tumour diameter (cm) and the number of tumours (TBS^2^ = maximum diameter^2^ + number of tumours^2^), was calculated according to Sasaki et al. [8].

The Charlson Comorbidity Index [9] was used to describe patients’ comorbidities.

Liver resection, MWA, radiofrequency ablation (RFA), and percutaneous ethanol injection (PEI) performed for metastatic disease before study inclusion were classified as ‘liver-directed surgical procedures’. The broader term ‘liver-directed treatments’ was used when trans-arterial procedures were also included.

Radical-intent surgery was defined as a surgical procedure performed with the goal of complete ablation of hepatic tumour burden. Non-radical-intent surgery was defined as a procedure aiming to reduce morbidity and preserve liver function (e.g., debulking or palliative procedures).

Intraoperative complications were recorded and classified according to the Oslo classification [10]. Post-MWA complications were assessed within 30 days after surgery; complications were recorded as follows: fever (requiring prolongation or change in antimicrobial therapy), nausea and vomiting, pleural effusion (if treated with albumin infusion and diuretic therapy or thoracentesis), pneumothorax (if pleural drainage was needed), ascites (requiring albumin supplementation or diuretics), haemoperitoneum, and liver function impairment according to the 50-50 criteria [11]. Safety was graded using the Clavien–Dindo classification [12] and the comprehensive complication index (CCI) [13]. A CCI ≥ 26.2 was used as the cutoff for severe postoperative morbidity after liver surgery [14]. Post-MWA mortality was recorded as death during hospitalisation or within 30 and 90 days after the procedure. Readmission due to any treatment-related complication was recorded within 30 days after MWA. Prolonged length of hospital stay (LOS) was defined as discharge later than 3 days [1].

Contrast-enhanced CT or MRI was repeated one month after MWA to assess the efficacy of ablation: the disappearance of any intra-tumoural arterial enhancement in the target lesion(s) defined a complete response (CR) [15].

Analyses were performed per patient and per procedure, considering every MWA procedure during follow-up.

The efficacy of the procedure was defined by the CR achieved in the target lesion and the absence of mortality within 90 days.

As recently proposed [1,16], the TO was defined as follows: absence of post-MWA complications, a hospital stay of ≤3 days, no mortality or re-admission within 30 days, and CR of the target lesion at 1-month post-MWA CT scan.

OS was calculated from the time of surgery to the last follow-up available or death. Non-evidence of disease (NED) was defined as the absence of disease at the patient’s last available follow-up or death.

The authors are accountable for all aspects of the work in ensuring that questions related to the accuracy or integrity of any part of the work are appropriately investigated and resolved. The study was conducted in accordance with the Declaration of Helsinki (as revised in 2013). Data were collected and managed in accordance with GDPR 2016/679. Each patient gave written consent for every procedure performed and for the use of data for research and publication purposes. All procedures were performed in accordance with the Declaration of Istanbul. No one received compensation or was offered any incentive for participating in this study. The study was approved by the Territorial Ethics Committee Central—Eastern Veneto Area (CET—ACEV) (protocol number 6344/A0/25, 12 June 2025).

### Statistical Analysis

Values for categorical variables were expressed as totals and percentages, whereas for continuous variables they were expressed as medians and interquartile ranges (IQRs). Statistical analyses were performed using Pearson’s chi-square test or Fisher’s exact test for categorical variables and the Wilcoxon rank-sum test for continuous variables. The length of follow-up was calculated from the date of surgery to the date of the patient’s latest follow-up date. The duration of follow-up was expressed as median (IQR). OS and recurrence curves were calculated using the Kaplan–Meier technique and compared with the log-rank test. Factors associated with OS were evaluated using univariable and multivariable Cox proportional-hazards models, reporting hazard ratios (HRs) with 95% confidence intervals (95% CIs). Factors associated with efficacy were evaluated using univariable and multivariable logistic regression, reporting odds ratios (ORs) with 95% CIs. A *p*-value < 0.05 indicated statistical significance; variables with a *p*-value < 0.1 were considered of marginal statistical significance. Statistical analyses were performed using R/RStudio 4.5 (2025).

## 3. Results

**Figure 1:**
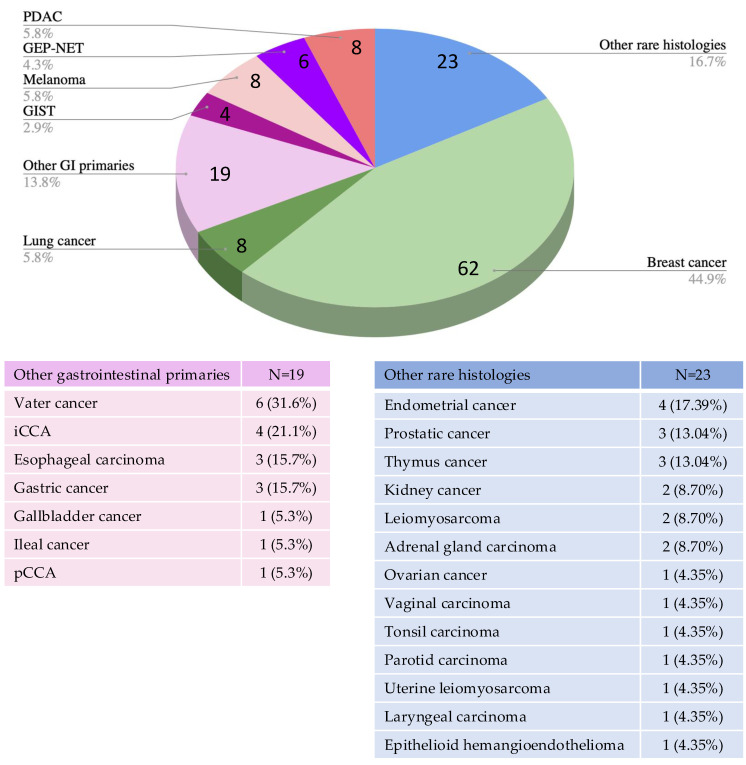
Category of liver metastases according to the primary tumours. PDAC, pancreatic ductal adenocarcinoma; GIST, gastrointestinal stromal tumour; GEP-NET, gastro-entero-pancreatic neuroendocrine tumour; GI gastrointestinal.

Between January 2010 and December 2024, 138 patients underwent 172 MWA procedures eligible for the study. One hundred (72%) were female, and the median age was 61.5 (51.0, 70.0) years. Although only a negligible proportion of patients had a history of liver parenchyma disease, the median Charlson Comorbidity Index was as high as 8.0 (6.0, 9.0), with just two (1.5%) patients scoring ≤ 5. The median follow-up was 24.9 (10.3, 55.8) months. The main indication for ablation was breast cancer liver metastasis in 62 (44.9%) patients. The primary cancer pathology and other features of the study population are described in Figure 1 and Table 1.

One hundred and ten procedures used a minimally invasive video-assisted approach, the vast majority laparoscopic; two (1.2%) involved thoracoscopic transdiaphragmatic liver ablation. The concurrent procedures performed with the ablation are detailed in Table S1.

Tumour burden was significantly lower for percutaneous procedures. A history of previous liver-directed treatment was recorded before 38 (34.5%) video-assisted and 27 (43.5%) percutaneous procedures, exceeding one-third of procedures in both groups. Video-assisted procedures were significantly longer than percutaneous ones, likely owing in part to the treatment of a greater number of lesions and to the concurrent surgical procedures more frequently performed in this group (Table 2). The CR rate was significantly higher for video-assisted than percutaneous procedures (87% vs. 58%; *p* < 0.001). Perioperative characteristics are detailed in Table 2.

Ablations were conducted with non-radical intent in 40 out of 172 (23.3%) procedures. Patients treated with radical intent had a significantly lower neoplastic burden than their non-radical counterparts, as demonstrated by lower TBS (3 (2.1, 4.1) vs. 4.1 (2.7, 6.3), *p* = 0.001), less frequent bilobar disease (18 (14%) vs. 14 (35%), *p* = 0.002), and extrahepatic metastasis (7 (5.3%) vs. 19 (48%), *p* <0.001). Perioperative characteristics stratified by surgical intent are detailed in Table S2.

An open conversion was needed in only one case (peritoneal adhesions), and no transfusions were required; one Oslo Grade I intraoperative complication was recorded.

The postoperative course was uneventful for 149 (86%) procedures, with severe postoperative complications in just three (1.8%) cases. A CCI ≥ 26.2 occurred in five (2.9%) procedures. One procedure required postoperative intensive care admission for delayed extubation. One patient died due to acute liver decompensation three days after surgery, corresponding to a 30-day mortality rate of 0.6%. Overall, three patients died within 3 months after surgery, corresponding to a 90-day mortality rate of 1.7%. The median LOS was 2.0 (1.0, 3.0) days. The main postoperative outcomes are summarized in Table 3.

The TO was achieved in 93 of 172 procedures (54%). Among the individual TO items, the CR of the target lesion had the lowest achievement rate (77%) and was therefore the main factor limiting TO attainment. This was not the case for video-assisted procedures, in which CR rates were higher and prolonged LOS became the principal limiting item instead. The proportion of procedures achieving each individual TO item—in the whole series and in the video-assisted and percutaneous subgroups—is shown in Figure S1.

**Figure 2:**
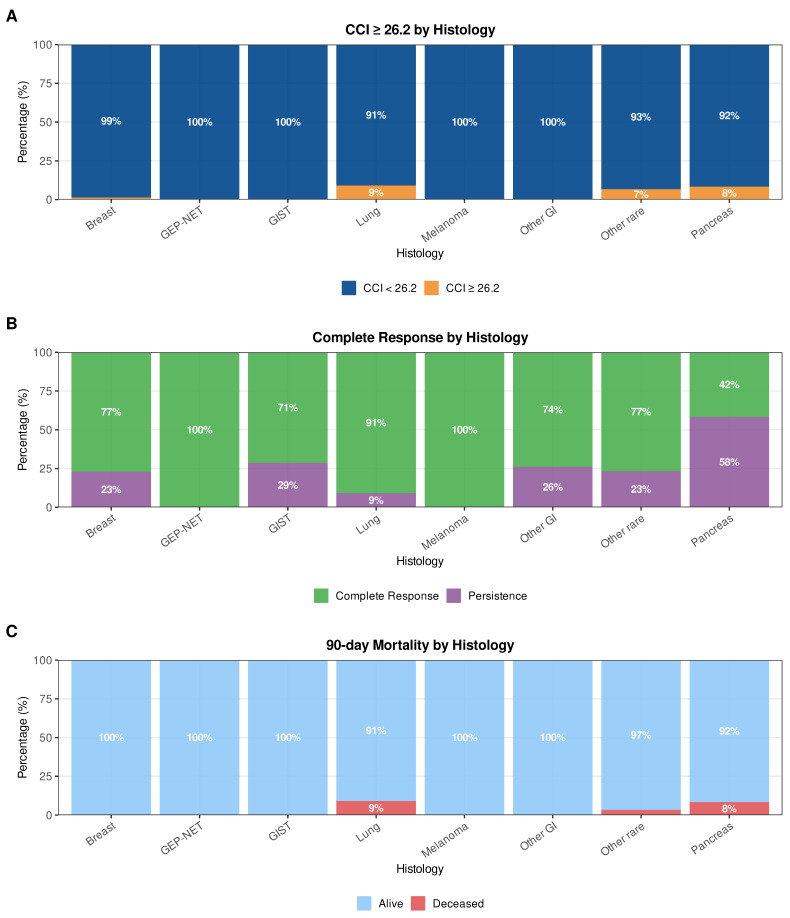
Outcome distribution by primary tumour histology. Stacked bar plots display the percentage of patients in each histological subgroup for three key outcomes: (**A**) proportion with a comprehensive complication index (CCI) ≥ 26.2, indicating high postoperative complication burden; (**B**) complete response vs. persistence; (**C**) 90-day mortality. Abbreviations: CCI, comprehensive complication index; GI, gastrointestinal; GIST, gastrointestinal stromal tumour; GEP-NET, gastro-entero-pancreatic neuroendocrine tumour.

The stacked bar plots in Figure 2 display the distribution of postoperative complication burden, CR, and 90-day mortality across primary tumour histologies.

### 3.1. Survival Analysis

**Figure 3:**
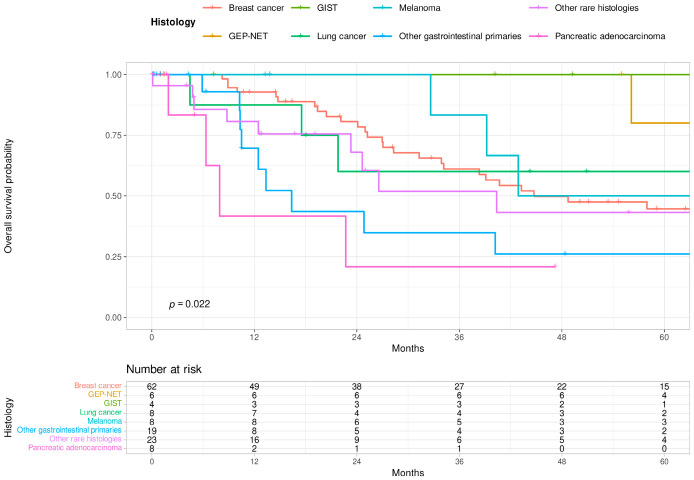
Kaplan–Meier survival curves of the study population stratified according to primary tumour histology.

OS was calculated per patient. The survival rate at 1, 3 and 5 years according to the tumour histology was as follows: breast cancer, 92.7%, 61.1%, and 44.7%, respectively; GIST, 100.0%, 100.0%, and 100.0%, respectively; lung cancer, 87.5%, 60.0%, and 60.0%, respectively; melanoma, 100%, 83.3%, and 50.0%, respectively; GEP-NET, 100.0%, 100.0%, and 80.0%, respectively; other gastrointestinal primaries, 69.6%, 34.8%, and 26.1%, respectively; other rare histologies, 80.6%, 51.8%, and 43.2%, respectively; and pancreatic adenocarcinoma, 41.7%, 20.8%, and 0.0%, respectively (Figure 3).

We then conducted a sub-analysis in those 104 patients who were treated with radical intent upfront. The survival rate at 1, 3 and 5 years according to the tumour histology was as follows: breast cancer, 92.9%, 68.8%, and 54.5%, respectively; GIST, 100.0%, 100.0%, and 100.0%, respectively; lung cancer, 83.3%, 83.3%, and 83.3%, respectively; melanoma, 100%, 80.0%, and 40.0%, respectively; GEP-NET, 100.0%, 100.0%, and 80.0%, respectively; other gastrointestinal primaries, 81.8%, 40.9%, and 30.7%, respectively; other rare histologies, 78.6%, 59.5%, and 59.5%, respectively; and pancreatic adenocarcinoma, 50.0%, 50.0%, and 0.0%, respectively (Figure S2).

Short- and long-term OS did not differ between procedures achieving TO and those that did not: the 1-, 3- and 5-year OS was 88.9%, 57.5% and 46.3% versus 82.2%, 56.3% and 38.4%, respectively (*p* = 0.32; Figure S3).

**Figure 4:**
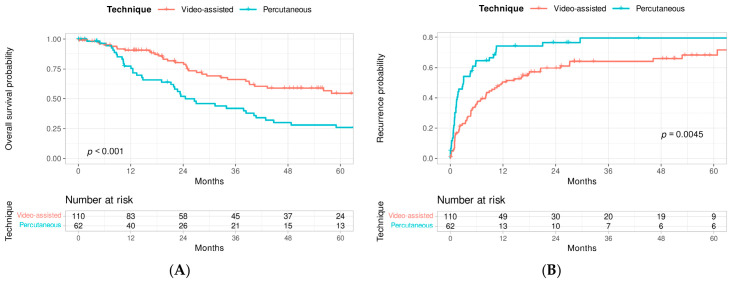
(**A**) Kaplan–Meier survival curves of MWA procedures stratified according to the surgical approach. (**B**) Kaplan–Meier recurrence probability curves of MWA procedures stratified according to the surgical approach.

By contrast, survival differed by surgical approach: the 1-, 3- and 5-year OS was 90.7%, 66.2% and 54.4% after the video-assisted procedure versus 77.5%, 41.9% and 26.0% after the percutaneous procedure (*p* = 0.00025; Figure 4A).

Recurrence occurred after 112 (65%) procedures, predominantly in the liver (Table S3).

**Figure 5:**
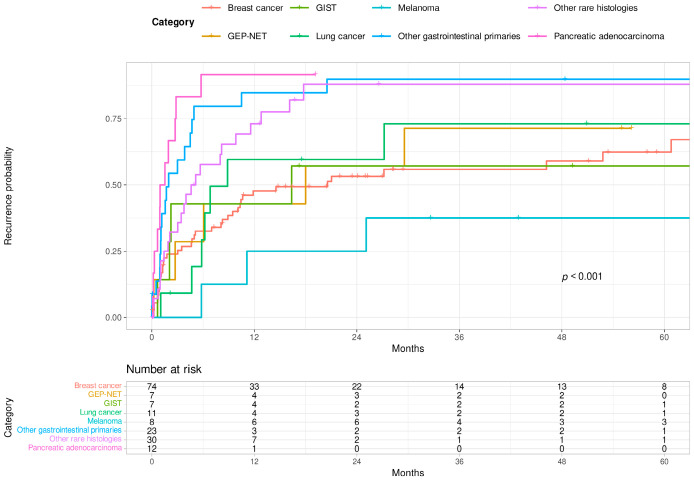
Kaplan–Meier recurrence probability curves of MWA procedures stratified according to primary tumour histology.

The recurrence probability was calculated per procedure. The recurrence rate at 1, 3 and 5 years according to the tumour histology was as follows: breast cancer, 47.7%, 55.8%, and 62.4%, respectively; GIST, 42.9%, 57.1%, and 57.1%, respectively; lung cancer, 59.6%, 73.1%, and 73.1%, respectively; melanoma, 25.0%, 37.5%, and 37.5%, respectively; GEP-NET, 42.9%, 71.4%, and not available, respectively; other gastrointestinal primaries, 84.7%, 89.9%, and 89.9%, respectively; other rare histologies, 73.1%, 88.0%, and 88.0%, respectively; and pancreatic adenocarcinoma 91.7%, not available, and not available, respectively (Figure 5).

The recurrence probability at 1, 3 and 5 years was 50.5%, 64.1% and 68.2% after video-assisted procedures versus 74.3%, 79.4% and 79.4% after percutaneous procedures (*p* = 0.0045; Figure 4B).

Surgery was the treatment of choice for recurrence in 55 out of 112 (49.1%) cases, with repeat MWA as the most frequent surgical modality (48 cases, 43%; Table S3).

At the end of the follow-up, 69 (50.0%) patients achieved NED, 11 (8.0%) of them after multiple recurrence treatments.

### 3.2. Factors Associated with Procedure Efficacy

One hundred and thirty (75.6%) procedures were effective according to our definition: the CR of the target lesion and absence of 90-day mortality. The univariable analysis recorded pancreatic adenocarcinoma primary (OR 0.20; 95% CI 0.06, 0.66; *p* = 0.009), ECOG PS ≥ 2 (OR 0,16; 95% CI 0.02, 0.84; *p* = 0.037), previous liver resection (OR 0.30; 95% CI 0.13, 0.73; *p* = 0.007), number of previous liver surgeries (OR 0.76; 95% CI 0.58, 0.98; *p* = 0.039), and percutaneous technique (OR 0.24; 95% CI 0.11, 0.48; *p* < 0.001) as factors significantly associated with reduced efficacy. Non-radical surgical intent (OR 0.43; 95% CI 0.20, 0.93; *p* = 0.030) was associated with lower odds of efficacy, whereas operative time showed a positive but minimal association (OR 1.01; 95% CI 1.00, 1.02; *p* = 0.013); both, however, have limited clinical relevance or are subject to considerable uncertainty (Table 4). In multivariable logistic regression, the percutaneous approach (OR 0.14, 95% CI 0.05–0.34, *p* < 0.001) versus the video-assisted approach, more than three nodules (OR 0.13, 95% CI 0.04–0.42, *p* < 0.001), ECOG PS ≥ 2 (OR 0.05, 95% CI 0.01–0.34, *p* = 0.003) and previous liver resection (OR 0.25, 95% CI 0.09–0.67, *p* = 0.006) were independently associated with lower odds of efficacy, whereas the histological prognostic group was not (Table 4).

### 3.3. Factors Associated with OS

Factors associated with OS were assessed by univariable and multivariable Cox proportional-hazards analyses (Table S4).

In the univariable analysis, worse OS was associated with ECOG PS ≥ 2 (HR 4.43, 95% CI 1.37–14.3, *p* = 0.013), the percutaneous approach (HR 2.08, 95% CI 1.25–3.45, *p* = 0.005), and non-radical surgical intent (HR 3.34, 95% CI 1.92–5.82, *p* < 0.001), with borderline associations for a higher TBS (HR 1.07, 95% CI 1.00–1.15, *p* = 0.058) and extrahepatic metastasis (HR 1.94, 95% CI 1.00–3.78, *p* = 0.050). Among individual primary histologies, pancreatic adenocarcinoma (HR 3.62, 95% CI 1.30–10.1, *p* = 0.014) and other GI primaries (HR 2.10, 95% CI 1.03–4.29, *p* = 0.042) were associated with worse survival, whereas the pre-defined histological prognostic group was not. Worse survival was further associated with adverse post-procedural events—severe complications (Clavien–Dindo ≥ 3, HR 4.09, 95% CI 1.29–13.0, *p* = 0.017), 30-day readmission (HR 3.45, 95% CI 1.22–9.76, *p* = 0.019), and incomplete local response (no CR, HR 2.04, 95% CI 1.17–3.55, *p* = 0.012)—whereas achievement of efficacy was protective (HR 0.45, 95% CI 0.26–0.78, *p* = 0.005) (Table S4).

In the multivariable Cox analysis, the percutaneous approach (HR 2.44, 95% CI 1.38–4.31, *p* = 0.002) versus the video-assisted approach, ECOG PS ≥ 2 (HR 6.06, 95% CI 1.78–20.7, *p* = 0.004) and the TBS (HR 1.09 per unit, 95% CI 1.01–1.18, *p* = 0.032) were independently associated with worse OS, whereas the histological prognostic group and extrahepatic metastasis were not (Table S4).

## 4. Discussion

In this single-centre retrospective analysis of 138 patients who underwent 172 MWA procedures for NCRLM over a 14-year period, we evaluated the safety and efficacy of thermal ablation in a heterogeneous cohort spanning eight primary tumour histologies. Three main findings emerged. First, MWA was associated with a favourable safety profile, with a low rate of severe complications and a 30-day mortality below 1%. Second, oncological efficacy was achieved in roughly three-quarters of procedures and was driven mainly by the CR rate, which was markedly higher in the video-assisted than in the percutaneous approach. Third, five-year OS was profoundly influenced by primary tumour biology, ranging from the most favourable histologies (GIST and GEP-NET) to a dismal outcome in pancreatic adenocarcinoma. These histology-specific differences, although biologically plausible, should be interpreted with caution, as the small size of several subgroups limits the stability and generalisability of the corresponding survival estimates. Collectively, these findings support MWA as a versatile and repeatable locoregional modality whose clinical value is modulated by technical approach, patient performance status, tumour burden, and the biological behaviour of the primary tumour.

The safety profile observed in our series compares favourably with published experiences in the thermal ablation of liver malignancies. In the Phase III COLLISION trial, which compared thermal ablation with hepatic resection for colorectal liver metastases, van der Lei and colleagues reported zero procedure-related mortality and a significantly lower complication burden in the ablation arm than in the resection arm [17]. Similarly, in the setting of neuroendocrine liver metastases, Frilling and colleagues reported RFA-related morbidity in approximately 5% of patients and no procedure-related mortality [18]. In their recent comparative review of ablative therapies for hepatic metastases, Torielli et al. described radiofrequency and microwave ablation as procedures whose complication profiles are dominated by minor adverse events—pain, infection, bleeding, and rare thermal injury to adjacent structures—without explicitly quantifying procedure-related mortality in the pooled literature [19]. Our Clavien–Dindo ≥ 3 rate of 1.8% and 30-day mortality of 0.6% are therefore consistent with these benchmarks. The use of two complementary and independent metrics—the Clavien–Dindo classification, which identifies the worst single complication, and the CCI, which aggregates the cumulative burden of all postoperative events—provides a more granular assessment of safety than either instrument alone. Because a CCI ≥ 26.2 represents the validated threshold for severe postoperative morbidity after hepatobiliary surgery [14], the low proportion of procedures reaching this cutoff in our series (2.9%) directly reflects the overall safety of MWA. This profile is particularly relevant in a NCRLM population characterised by substantial comorbidities (73% of our cohort had a Charlson Comorbidity Index ≥ 7), where repeatability and minimal physiological cost are essential to support iterative locoregional strategies over extended disease trajectories.

When evaluated through the composite endpoint adopted in this study, the two components behaved asymmetrically: 90-day mortality was exceedingly rare, while the CR of the target lesion emerged as the dominant determinant of efficacy and was strongly modulated by surgical approach, being markedly higher after video-assisted than percutaneous procedures (*p* < 0.001). Accordingly, the percutaneous route persisted as the single strongest modifiable predictor of reduced efficacy in both the univariable and multivariable analyses (Table 4). These figures are aligned with our institutional experience with HCC, where laparoscopic MWA has been shown to deliver high rates of radical ablation and TO, particularly in lesions unfavourably located or difficult to access percutaneously [1,3]. The TO rate of 54% observed in the overall series should be interpreted in light of its limiting items (Figure S1): the incomplete CR of the target lesion was the single most frequent cause of failure, followed by prolonged LOS and post-ablation complications. TO achievement was nonetheless not associated with OS in the present cohort. The principal explanation is that, in patients with near-normal liver parenchyma, survival is governed primarily by tumour-related factors (type of primary tumour and TBS) and performance status, rather than by the peri-procedural quality that TO predominantly captures. Although individual TO components were associated with survival in the univariable analysis, the composite also incorporated prognostically neutral elements—notably prolonged LOS, the second most frequent cause of TO failure—so that, in a cohort of this size, the overall measure did not discriminate survival. This contrasts with the cirrhotic HCC setting, where the peri-procedural components co-vary with liver-function severity—itself a major determinant of survival—and TO therefore retains prognostic value [1,16].

Comparisons with previously published series should be interpreted with caution, as cohorts differ substantially in patient selection, in the distribution of primary tumour histologies (e.g., colorectal, neuroendocrine, and mixed non-colorectal cohorts) and in the ablation technique; such heterogeneity limits the direct comparability of efficacy, recurrence, and survival outcomes across studies.

The univariable analysis of factors associated with efficacy identified five variables that significantly reduced the probability of achieving the composite endpoint: pancreatic adenocarcinoma primary (OR 020; 95% CI 0.06, 0.66; *p* = 0.009), ECOG PS ≥ 2 (OR 0.16; 95% CI 0.02–0.84; *p* = 0.037), previous liver resection (OR 0.30; 95% CI 0.13–0.73; *p* = 0.007), number of previous liver surgeries (OR 0.76; 95% CI 0.58–0.98; *p* = 0.039), and percutaneous approach (OR 0.24; 95% CI 0.11–0.48; *p* < 0.001). Two additional variables —surgical intent (non-radical OR 0.43; 95% CI 0.20–0.93; *p* = 0.030) and operative time (OR 1.01; 95% CI 1.00–1.02; *p* = 0.013)—were associated with efficacy but warrant cautious interpretation. The lower efficacy observed with non-radical intent (equivalently, the higher efficacy with curative intent) is biologically coherent but reflects a pre-selection bias, as patients with lower tumour burden, unilobar disease, and absence of extrahepatic metastases were preferentially treated with curative intent (Table S2); it therefore describes the consequence of appropriate patient selection rather than an independent technical determinant of efficacy. The association with operative time is quantitatively minimal (OR 1.01 per minute) with a confidence interval that approaches unity, implying that clinical relevance is negligible despite statistical significance; this likely reflects the greater complexity—and therefore longer duration—of video-assisted procedures, which in turn are those with higher CR rates. Both observations illustrate the interpretive limits of univariable analysis in a heterogeneous cohort: in the multivariable model, these collinear variables were not retained, and efficacy was independently predicted by the surgical approach, the number of nodules, performance status, and previous liver resection. Prospective studies remain warranted to fully disentangle technical, oncological, and patient-level determinants of ablation efficacy.

Five-year OS varied widely according to primary tumour histology (Figure 3), and these gradients are concordant with the biology of each primary and with the available literature. Frilling and colleagues reported a 5-year OS of 53% after RFA for neuroendocrine liver metastases, a figure very close to our GEP-NET result and reflecting the indolent disease course of well-differentiated neuroendocrine neoplasms [18]. Narayanan and colleagues, in a multi-centre evaluation of ablation for breast cancer liver metastases, reported a 1- and 2-year OS of 90.1% and 55.9%, respectively [20]; our breast cancer cohort—the largest subgroup in the present series, accounting for 44.9% of patients—showed a comparable 1-year OS (92.7%) and 5-year OS of 44.7%, consistent with the natural history of a systemic disease where liver-directed therapy must be integrated within multimodal oncological care. At the opposite end of the biological spectrum, the uniformly poor survival of patients with pancreatic adenocarcinoma metastases calls into question the appropriateness of locoregional ablation in this setting and argues for extremely selective indications, ideally within dedicated clinical trials. The 2023 Appropriate Use Criteria issued by Hallemeier and colleagues on behalf of the American Radium Society support liver-directed therapy across selected non-colorectal primaries and provide a useful framework within which our histology-stratified outcomes can be interpreted [21]. Our histology-stratified data offer empirical corroboration of this framework, while also refining it: the striking contrast between the sustained survival observed in GIST and GEP-NET patients and the uniformly dismal outcomes in pancreatic adenocarcinoma suggests that future iterations of such recommendations should move beyond a binary “colorectal vs. non-colorectal” dichotomy toward a more granular, histology-specific stratification of appropriateness.

A direct comparison of MWA with the established treatment standards—surgical resection and stereotactic body radiation therapy (SBRT)—is methodologically constrained: unlike colorectal liver metastases, NCRLMs lack a consolidated, histology-specific standard of care, and no randomised trial has compared these modalities in this population, so cross-study comparison remains the only available approach and must be interpreted in light of the case-mix heterogeneity noted above. Surgical resection remains the reference treatment for resectable disease, but its benefit in the non-colorectal non-neuroendocrine setting is strongly histology-dependent and rests almost entirely on retrospective series, with a 5-year overall survival of approximately 36% in the largest multi-centre analysis of 1452 patients and wide variation according to primary tumour and prognostic profile [22], in line with the surgical literature we have already discussed [4,5]. These figures fall within the range spanned by our histological subgroups (5-year OS from 0% in pancreatic adenocarcinoma to 80–100% in GEP-NET and GIST), suggesting that MWA can achieve survival comparable to resection in appropriately selected patients while avoiding the morbidity of hepatectomy—a consideration of particular weight in a cohort where 73% of patients carried a Charlson Comorbidity Index ≥ 7. This interpretation is reinforced by higher-level evidence in colorectal metastases: thermal ablation proved non-inferior to resection for small lesions in the randomised COLLISION trial, with fewer complications and shorter hospital stay [17], while microwave ablation specifically yielded a 3-year overall survival of 78% versus 76% for resection in the prospective, propensity-matched MAVERRIC study [23]; although not directly transposable to NCRLMs, these data support the conceptual legitimacy of MWA as a primary, rather than merely salvage, locoregional option.

SBRT likewise offers high local control with minimal toxicity—a 2-year local control of 92% (100% for lesions ≤ 3 cm) in the multi-institutional phase I/II trial by Rusthoven et al. [24] and a 1-year local control of 87% with grade ≥ 3 toxicity in only 3.9% of 515 patients in the Dutch–Belgian registry [25]—provided an ablative biologically effective dose (BED_10_ ≥ 100 Gy) is delivered [26]. Direct comparisons between ablation and SBRT, however, are confined to colorectal cohorts, employ radiofrequency rather than microwave ablation, and are confounded by the preferential allocation of SBRT to lesions unsuitable for ablation [27]. Importantly, the efficacy of SBRT is modulated by tumour radiosensitivity, which differs markedly across histologies—colorectal, gastrointestinal stromal and melanoma metastases being comparatively radioresistant, whereas breast and neuroendocrine lesions are more radiosensitive [28]—and by molecular alterations such as KRAS mutation that further predict local failure [29]. Thermal ablation, by contrast, exerts a biology-independent cytotoxic effect, consistent with our own observation that the histological prognostic group did not independently predict ablation efficacy, and may be potentially advantageous in the radioresistant histologies well-represented within the NCRLM spectrum. Taken together, the available evidence does not establish the superiority of any single modality but rather positions surgical resection, thermal ablation and SBRT as complementary tools to be selected within a multidisciplinary framework according to tumour location, number and size, hepatic and extrahepatic burden, performance status, and primary tumour biology [19,21,30]. Within this framework, the principal value of MWA lies less in replacing resection or SBRT than in offering a parenchyma-sparing and inherently repeatable platform—directly demonstrated in our series, where repeat MWA was the most frequent treatment for first hepatic recurrence and contributed to a final non-evidence-of-disease rate of 50%—that can be integrated with systemic therapy across the extended disease trajectory typical of metastatic non-colorectal cancer.

The video-assisted approach was associated with superior OS and a lower recurrence probability at all time points. This advantage persisted after multivariable adjustment for the histological prognostic group, TBS, extrahepatic disease, and performance status: the percutaneous approach remained independently associated with worse OS (HR 2.44, 95% CI 1.38–4.31, *p* = 0.002), with the effect estimate, if anything, strengthened relative to the unadjusted comparison—indicating that the survival difference is not explained by the measured baseline imbalances between the two groups. In the same model, an ECOG PS ≥ 2 (HR 6.06, 95% CI 1.78–20.7) and a higher TBS (HR 1.09 per unit, 95% CI 1.01–1.18) were the other independent predictors of survival, whereas neither the histological prognostic grouping nor extrahepatic disease retained independent significance; once burden, fitness and approach are accounted for, the grouped primary histology thus carries no independent prognostic weight, despite the strong univariable signal of aggressive histologies such as pancreatic adenocarcinoma. In our centre, the choice between video-assisted and percutaneous MWA follows institutional criteria developed over more than two decades of HCC experience [3,6], whereby laparoscopic ablation is preferred when the percutaneous route is not feasible or when concurrent procedures are indicated—circumstances markedly more frequent in the video-assisted group (Table 2). Notably, the liver-function criteria embedded in the original HCC selection algorithm had little bearing on the present cohort, in which chronic liver disease was uncommon (cirrhosis 2.9%, CSPH 1.4%); approach allocation was therefore governed almost entirely by the technical and anatomical criteria described in the Materials and Methods. Consistently, anatomically favourable, lower-burden lesions amenable to percutaneous treatment were preferentially allocated to that route, accounting for the lower tumour burden observed in the percutaneous cohort. The video-assisted approach allows for direct visualisation of the liver surface, intraoperative ultrasound with higher spatial resolution, active protection of adjacent organs, and the simultaneous treatment of multiple nodules (median 2 vs. 1, *p* = 0.001). These factors likely contribute to the higher CR rate and, in turn, to improved long-term disease control. Notably, the video-assisted approach remained independently associated with efficacy after adjustment for tumour burden, histological prognostic group, performance status, and previous liver resection, indicating that the higher efficacy of this approach is not explained by the measured baseline imbalances. A plausible mechanism links the two endpoints: the video-assisted approach achieved higher rates of CR and efficacy (Table 4), and incomplete local response was itself associated with worse OS in the univariable analysis (no CR, HR 2.04, 95% CI 1.17–3.55); the survival benefit of the video-assisted approach therefore appears to be mediated, at least in part, by improved local tumour control. Residual confounding by unmeasured factors—in particular lesion location, which was not captured as a structured variable—cannot, however, be excluded, and prospective or propensity-score-matched studies remain warranted to confirm these findings.

This study has several limitations. First, its retrospective single-centre design carries inherent selection bias. Second, the histological heterogeneity of the cohort—while reflecting the real-world case-mix of a tertiary hepatobiliary unit—limits the statistical power of histology-specific survival analyses, particularly for smaller subgroups (e.g., GIST, melanoma, pancreatic adenocarcinoma, and the rare histologies); the corresponding per-histology survival and recurrence estimates should therefore be regarded as exploratory and hypothesis-generating rather than definitive, and for this reason, the multivariable analyses were based on a pre-defined histological prognostic grouping rather than on individual histologies. Third, although multivariable models were performed for both efficacy and OS, the limited number of events required parsimonious, pre-specified models and the exclusion of collinear and post-procedural variables; larger, multi-centre cohorts would allow more comprehensive modelling and external validation. Fourth, our safety definition, while methodologically robust, may not capture the full burden of minor or functional sequelae relevant to patient-reported outcomes; similarly, treatment-free interval and treatment-free survival—potentially meaningful quality-of-life endpoints—could not be reliably assessed within the present dataset and warrant evaluation in future prospective studies. Finally, the comparison between video-assisted and percutaneous approaches reflects institutional allocation criteria, including a different distribution of primary tumour histologies and a numerically higher proportion of radical-intent treatment in the video-assisted group (Table 2); although this comparison was adjusted for the principal measured confounders by a multivariable analysis, residual confounding by unmeasured factors cannot be excluded. In particular, the anatomical location of treated lesions was not captured as a structured variable, precluding quantification of the proportion of lesions in unfavourable/critical sites and a formal assessment of its role in approach allocation.

Despite these limitations, the size of the cohort, the 14-year time frame, the breadth of histologies represented, and the adoption of contemporary safety and efficacy definitions provide a comprehensive portrait of MWA as currently practised for NCRLMs at a high-volume European hepatobiliary centre, and identify the video-assisted approach, patient performance status, and primary tumour biology as the key axes around which future prospective evaluations should be structured.

## 5. Conclusions

In this 14-year single-centre experience, MWA emerged as a safe and oncologically effective locoregional treatment for NCRLMs. The video-assisted approach was associated with substantially higher CR rates and superior long-term survival than the percutaneous route, though these differences likely reflect the combined effect of patient selection, technical completeness, and the opportunity to perform concurrent procedures. Five-year OS varied widely across primary tumour histologies; in the multivariable analysis, the independent determinants of survival were tumour burden, performance status, and the procedural approach, rather than the histological category in isolation—indicating that outcomes reflect the interplay of tumour biology, disease burden, patient fitness, and treatment delivery. Within this framework, MWA offers a repeatable, minimally invasive platform for iterative locoregional disease control—often aimed at avoiding tumour-related liver morbidity and mortality or at prolonging the interval between chemotherapy cycles, most effective when integrated into multidisciplinary treatment pathways and calibrated to the specific histology, performance status, and prior treatment history of each patient. Prospective, histology-stratified studies are warranted to refine patient selection and to disentangle the independent contributions of technique and biology to long-term outcomes.

## Figures and Tables

**Table 1:** Patients’ demographics and characteristics.

Variables	N = 138 ^1^
Age (years)	61.5 (51.0, 70.0)
Sex (female)	100 (72%)
ECOG PS ≥ 2 *missing	4 (3.1%) *10
CCI ≥ 7 *missing	98 (73%) *4
Diabetes (yes) *missing	12 (8.8%) *1
Cirrhosis (yes)	4 (2.9%)
HCV positive	1 (0.7%)
NAFLD	2 (1.4%)
CSPH	2 (1.4%)
Last patient status	
Died with recurrence	52 (38%)
Died without recurrence	38 (28%)
Alive with disease	17 (12%)
Alive without disease	20 (14%)
Alive without disease after last recurrence treatment	11 (8.0%)
Median follow-up (months)	24.9 (10.3, 55.8)

^1^ Median (Q1, Q3); n/N (%); ECOG PS, Eastern Cooperative Oncology Group Performance Status; CCI, Charlson Comorbidity Index; HCV, hepatitis C virus; NAFLD, non-alcoholic fatty liver disease; CSPH, clinically significant portal hypertension; * missing values.

**Table 2:** Perioperative characteristics of video-assisted vs percutaneous procedures.

Variable	Video-Assisted N = 110 ^1^	Percutaneous N = 62 ^1^	*p* ^2^
**Category of the Primary Tumours**			
Breast cancer	49 (45%)	25 (40%)	0.19
GEP-NET	6 (5.5%)	1 (1.6%)	
GIST	6 (5.5%)	1 (1.6%)	
Lung	9 (8.2%)	2 (3.2%)	
Melanoma	4 (3.6%)	4 (6.5%)	
Other GI primaries	14 (13%)	9 (15%)	
Other rare histologies	18 (16%)	12 (19%)	
Pancreatic adenocarcinoma	4 (3.6%)	8 (13%)	
**Preoperative variables**			
Platelets (10^9^/L) *missing	208 (165.0, 267.0) *3	213 (166.0, 277.0) *7	0.81
Bilirubin (umol/L) *missing	10.4 (7.4, 16.4) *6	10 (6.9, 13.5) *11	0.23
Nodules (n)	2 (1.0, 3.0)	1 (1.0, 2.0)	0.001
Major diameter	2.3 (1.6, 3.2)	1.9 (1.4, 3.5)	0.36
TBS	3.4 (2.4, 5.2)	2.7 (1.8, 4.3)	0.009
Bilobar disease	22 (20%)	10 (16%)	0.53
Major vessel invasion	1 (0.9%)	0 (0%)	>0.99
Extrahepatic metastasis	14 (13%)	12 (19%)	0.24
Previous liver-dir. surgery			
• None	76 (69%)	37 (60%)	0.27
• 1	21 (19%)	17 (27%)	
• 2–4	11 (9.9%)	6 (9.6%)	
• ≥5	2(1.8%)	2 (3.2%)	
Previous liver-dir. treatments			0.25
• None	72 (65.5%)	35 (56.5%)	
• 1	23 (20.9%)	19 (30.7%)	
• 2–4	12 (10.9%)	5 (8%)	
• ≥5	3 (2.7%)	3 (4.8%)	
**Intraoperative variables**			
Radical Intent	89 (81%)	43 (69%)	0.085
Concurrent procedure	48 (44%)	3 (4.8%)	<0.001
Operative time (min) *missing	100 (80.0, 125.0) *2	25 (15.0, 32.5) *2	<0.001
Nodules treated *missing	2 (1.0, 3.0) *2	1 (1.0, 1.0) *2	<0.001
Duration, ∑ (min) *missing	12 (8.0, 18.0) *2	7 (5.0, 10.0) *1	<0.001
Power (Watt)	40 (40.0, 60.0)	40 (40.0, 60.0)	0.062
**Postoperative variables**			
LOS	2.5 (2.0, 3.0)	1 (1.0, 1.0)	<0.001
Postoperative complications	21 (19%)	2 (3.2%)	0.003
Reoperation	2 (1.8%)	0 (0%)	0.54
Clavien–Dindo ≥ 3	3 (2.7%)	0 (0%)	0.70
CCI ≥ 26.2	5 (4.5%)	0 (0%)	0.16
CR of the target lesion	96 (87%)	36 (58%)	<0.001
TO (achieved)	61 (55%)	32 (52%)	0.63
90-day mortality	2 (1.8%)	1 (1.6%)	>0.99

^1^ n/N (%); median (Q1, Q3); ^2^ Pearson’s chi-square test; Fisher’s exact test; Wilcoxon rank-sum test; NA. GEP-NET, gastro-entero-pancreatic neuroendocrine tumour; GIST, gastrointestinal stromal tumour; GI, gastrointestinal; TBS, tumour burden score; MWA, microwave ablation; PEI, percutaneous alcohol injection; RFA, radiofrequency ablation; TACE, trans-arterial chemoembolization; LOS, length of hospital stay; CCI, comprehensive complication index; CR, complete response; TO, textbook outcome; * missing values.

**Table 3:** Postoperative outcomes.

Variable	N (%)
LOS > 3	27 (15.6%)
Fever	13 (7.6%)
Nausea and vomiting	7 (4.1%)
Pleural effusion	2 (1.2%)
Pneumothorax	0 (0.0%)
Ascites	1 (0.6%)
Liver function impairment (50-50 criteria)	1 (0.6%)
Haemoperitoneum	1 (0.6%)
Readmission within 30 days	9 (5.2%)
30 days mortality	1 (0.6%)
No CR	40 (23%)

LOS, length of hospital stay; CR, complete response.

**Table 4:** Univariable and multivariable analyses of factors associated with efficacy.

	Univariable			Multivariable		
Variable	OR	95% CI	*p*	OR	95% CI	*p*
Breast cancer	1.15	0.57, 2.36	0.7			
GIST	0.80	0.17, 5.74	0.8			
Lung cancer	1.49	0.36, 10.0	0.6			
Other gastrointestinal primaries	0.90	0.35, 2.66	0.8			
Other rare histologies	0.87	0.36, 2.23	0.8			
Pancreatic adenocarcinoma	0.20	0.06, 0.66	0.009			
Histological prognostic group *:						
• Favourable (ref.)	-	-	-	-	-	-
• Unfavourable	0.94	0.43, 2.07	0.9	1.17	0.47, 2.99	0.7
• Other	0.84	0.33, 2.29	0.7	0.96	0.32, 3.11	>0.9
Age	0.99	0.97, 1.02	0.7			
Sex (male)	0.89	0.42, 1.94	0.8			
ECOG PS ≥ 2	0.16	0.02, 0.84	0.037	0.05	0.01, 0.34	0.003
Number of nodules:						
• 1 (ref.)	-	-	-	-	-	-
• 2–3	1.02	0.46, 2.29	>0.9	0.75	0.28, 1.95	0.5
• > 3	0.40	0.16, 1.03	0.054	0.13	0.04, 0.42	<0.001
Major diameter	0.96	0.82, 1.15	0.6			
TBS	0.94	0.84, 1.04	0.2			
Bilobar disease	0.54	0.24, 1.28	0.15			
Extrahepatic metastasis	0.86	0.34, 2.34	0.7			
Previous liver resection	0.30	0.13, 0.73	0.007	0.25	0.09, 0.67	0.006
Previous Liver Ablation	0.77	0.37, 1.65	0.5			
Previous liver treatments	0.66	0.33, 1.35	0.3			
Number of previous liver surgery	0.76	0.58, 0.98	0.039			
Number of previous liver treatments	0.80	0.62, 1.02	0.066			
Surgical technique:						
• Video-assisted approach (ref.)	-	-	-	-	-	-
• Percutaneous approach	0.24	0.11, 0.48	<0.001	0.14	0.05, 0.34	<0.001
Concurrent procedure	1.47	0.68, 3.42	0.3			
Surgical Intent						
• Radical	-	-	-			
• Non-radical (ref.)	0.43	0.20, 0.93	0.030			
Operative time (min)	1.01	1.00, 1.02	0.013			
Nodules treated	0.98	0.81, 1.21	0.8			
Duration, ∑ (min)	1.02	0.97, 1.07	0.5			
Power (Watt)	1.01	0.99, 1.04	0.4			
LOS	1.09	0.90, 1.39	0.4			
Postoperative complications	1.63	0.57, 5.87	0.4			
Clavien–Dindo ≥ 3	0.56	0.09, 1.30	0.3			
CCI ≥ 26.2	1.30	0.19, 25.8	0.8			

* Histological prognostic group. Favourable: breast, GIST, GEP-NET; unfavourable: lung, melanoma, other gastrointestinal primaries, pancreatic adenocarcinoma; other: other rare histologies. CI, confidence interval; OR, odds ratio; GIST, gastrointestinal stromal tumour; GEP-NET, gastro-entero-pancreatic neuroendocrine tumour; ECOG PS, Eastern Cooperative Oncology Group Performance Status; TBS, tumour burden score; LOS, length of hospital stay; CCI, comprehensive complication index.

## Data Availability

The datasets generated during and/or analysed during the current study are not publicly available but are available from the corresponding author on reasonable request.

## Supplementary Materials

The following supporting information can be downloaded at: https://www.mdpi.com/article/10.3390/cancers18132026/s1, Figure S1: The proportion of procedures that achieved each desired health outcome forming the textbook outcome (TO) in the whole series, in the video-assisted approach, and in the percutaneous approach; Figure S2: Kaplan–Meier survival curves of patients who were treated with radical intent upfront, stratified according to primary tumour histology; Figure S3: Kaplan–Meier survival curves of MWA procedures stratified according to textbook outcome (TO) achievement; Table S1: Concurrent procedures fashioned with the ablation; Table S2: Perioperative characteristics of radical vs. non-radical intent procedures; Table S3: Recurrence pattern and treatments; Table S4: Univariable and multivariable analyses of factors associated with overall survival.

## Abbreviations

The following abbreviations are used in this manuscript:

BSC	Best Supportive Care
CCA	Cholangiocarcinoma
CCI	Comprehensive Complication Index
CR	Complete Response
CRLM	Colorectal Cancer Liver Metastases
CSPH	Clinically Significant Portal Hypertension
ECOG PS	Eastern Cooperative Oncology Group Performance Status
GEP-NET	Gastro-Entero-Pancreatic Neuroendocrine Tumour
GI	Gastrointestinal
GIST	Gastrointestinal Stromal Tumour
HCC	Hepatocellular Carcinoma
HCV	Hepatitis C Virus
ICU	Intensive Care Unit
LOS	Length of Hospital Stay
LR	Liver Resection
MWA	Microwave Ablation
NAFLD	Non-Alcoholic Fatty Liver Disease
NCRLM	Non-Colorectal Cancer Liver Metastasis
NED	Non-Evidence of Disease
OS	Overall Survival
PEI	Percutaneous Ethanol Injection
pRBC	Packed Red Blood Cells
RFA	Radiofrequency Ablation
TACE	Trans-Arterial Chemoembolization
