# Supplementary material for: Microwave Ablation for the Treatment of Non-Colorectal Cancer Liver Metastasis
Source: Cancers (Basel). 2026 Jun 23;18(13):2026. doi: 10.3390/cancers18132026 (PMC13360228; doi:10.3390/cancers18132026)
Supplement: Supplementary file 1 [file cancers-18-02026-s001.zip › cancers-4315671-supplementary.pdf]

cancers

MDPI

Article

Microwave Ablation for the Treatment of Non-Colorectal Cancer Liver Metastasis

Jacopo Lanari [1,2,*,†], Sara Lazzari [1,†], Ilaria Billato [3], Chiara Naldini [1], Clarissa De Nardi [1], Giulia Tamponi [1], Davide Volpato [1], Alessandro Furlanetto [1,2], Francesco Enrico D'Amico [1,2], Alessandro Vitale [1,2], Enrico Gringeri [1,2] and Umberto Cillo [1,2]

1 Department of Surgical, Oncological and Gastroenterological Sciences, University of Padua, 35128 Padua, Italy
2 General Surgery and Hepato-Pancreato-Biliary Surgery and Liver Transplantation Unit, Padua University Hospital, 35128 Padua, Italy
3 Department of Biology (DiBio), University of Padua, 35128 Padua, Italy
* Correspondence: jacopo.lanari@unipd.it; Tel.: +39-049-821-8601
† These authors contributed equally to this work.

Supplementary Material

Table S1. Concurrent procedures fashioned with the ablation

Concurrent procedure	N = 51[1]
Liver biopsy	26 (51%)
Cholecystectomy	8 (16%)
Lysis of peritoneal adhesions	7 (14%)
Liver biopsy and Cholecystectomy	3 (5.9%)
PEI	2 (3.9%)
Peritoneal biopsy	2 (3.9%)
Abdominal wall lesion excision	1 (2.0%)
Liver biopsy and unroofing hepatic cyst	1 (2.0%)
Liver biopsy and PEI	1 (2.0%)

[1] n / N (%).

PEI, percutaneous ethanol injection.

Table S2. Perioperative characteristics of radical vs. non-radical intent procedures

Variable	No Radical N = 40[1]	Radical N = 132[1]	p^2
Platelets (10^9/L) *missing	225 (177.0, 245.0) *3	206 (165.0, 277.0) *7	0.76
Bilirubin (umol/L) *missing	10.4 (7.0, 13.5) *6	10 (7.5, 16.3) *11	0.71
Nodules (n)	2 (1.0, 3.5)	1.5 (1.0, 2.0)	0.024
Major diameter	2.6 (1.6, 4.5)	2 (1.5, 3.0)	0.077
TBS	4.1 (2.7, 6.3)	3 (2.1, 4.1)	0.001
Bilobar disease	14 (35%)	18 (14%)	0.002
Major vassels invasion	1 (0.6%)	0 (0%)	0.23
Extrahepatic metastasis	19 (48%)	7 (5.3%)	<0.001
Previous liver-dir. surgery			
• None	30 (75%)	83 (63%)	0.42
• 1	7 (18%)	31 (23%)	
• 2 - 4	3 (7.5%)	14 (10.6%)	
• ≥5	0 (0%)	4 (3.1%)	
Previous liver-dir. treatments			
• None	29 (73%)	78 (59%)	0.28
• 1	8 (20%)	34 (26%)	
• 2 - 4	2 (5.0%)	15 (11.3%)	
• ≥5	1 (2.5%)	5 (3.8%)	
**Intra-operative variables**			
Surgical technique			
• Video-assisted	21 (53%)	89 (67%)	0.085
• Percutaneous	19 (48%)	43 (33%)	
Operative time (min)*missing	70 (20.0, 100.0) *1	80 (35.0, 115.0) *3	0.18
Nodules treated *missing	1 (1.0, 2.0) *2	1 (1.0, 2.0) *2	0.48
Duration, Σ (min) *missing	10 (7.0, 15.0)	10 (6.0, 14.0) *3	0.59
Power (Watt)	40 (40.0, 60.0)	40 (40.0, 60.0)	0.062
**Post-operative variables**			
LOS	2 (1.0, 3.0)	2 (1.0, 3.0)	0.23
Postoperative complications	5 (13%)	18 (14%)	0.85
Reoperation	0 (0%)	2 (1.5%)	>0.99
Clavien-Dindo ≥ 3	1 (2.5%)	2 (1.5%)	0.27
CCI ≥ 26.2	1 (2.5%)	4 (3.0%)	>0.99
CR of the target lesion	26 (65%)	106 (80%)	0.045
TO (achieved)	19 (48%)	74 (56%)	0.34

[1] n / N (%); Median (Q1, Q3).

[2] Pearson’s Chi-squared test; Fisher’s exact test; Wilcoxon rank sum test; NA.

* missing values

TBS, tumour burden score; MWA, microwave ablation; PEI, percutaneous alcohol injection; RFA, radiofrequency ablation; TACE, trans-arterial chemoembolization; pRBC, packed red blood cells; ICU, intensive care unit; LOS, length of hospital stay; CCI, comprehensive complication index; CR, complete response;TO, Textbook outcome.

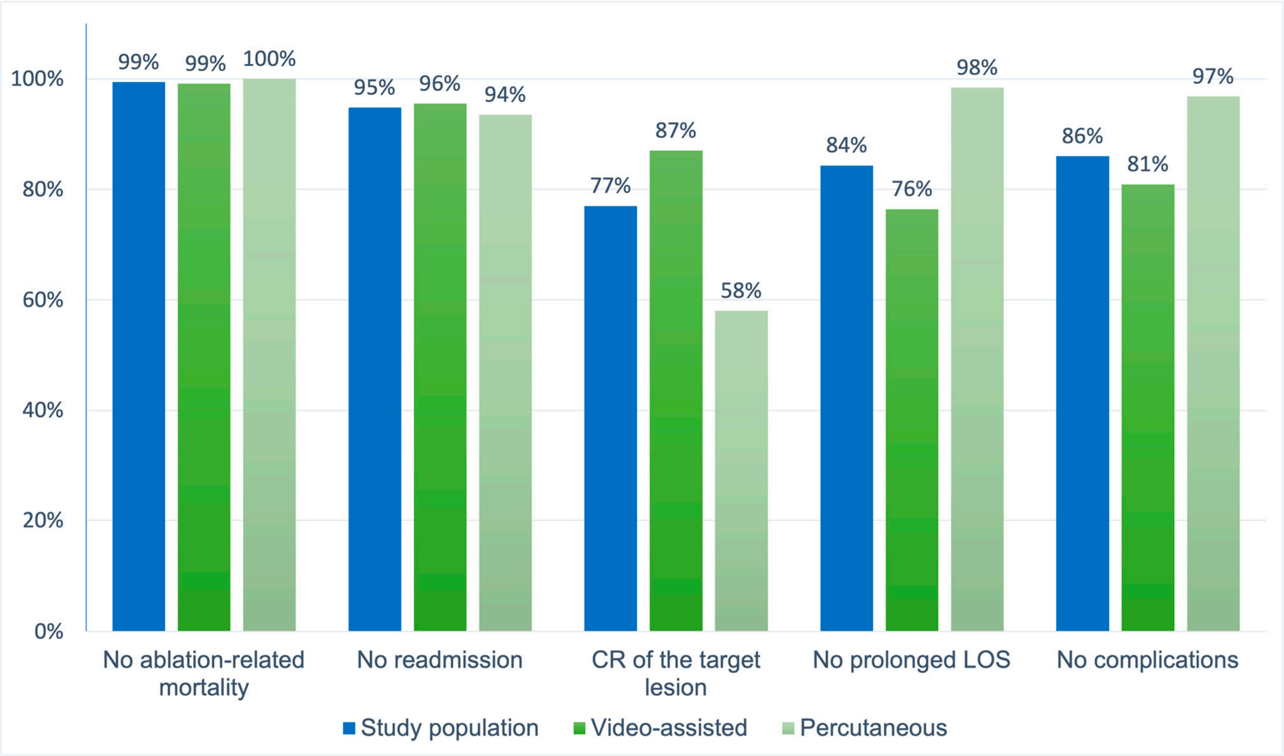

Figure S1. The proportion of procedures that achieved each desired health outcome forming the textbook outcome (TO) in the whole series, in the video-assisted, and percutaneous approach.

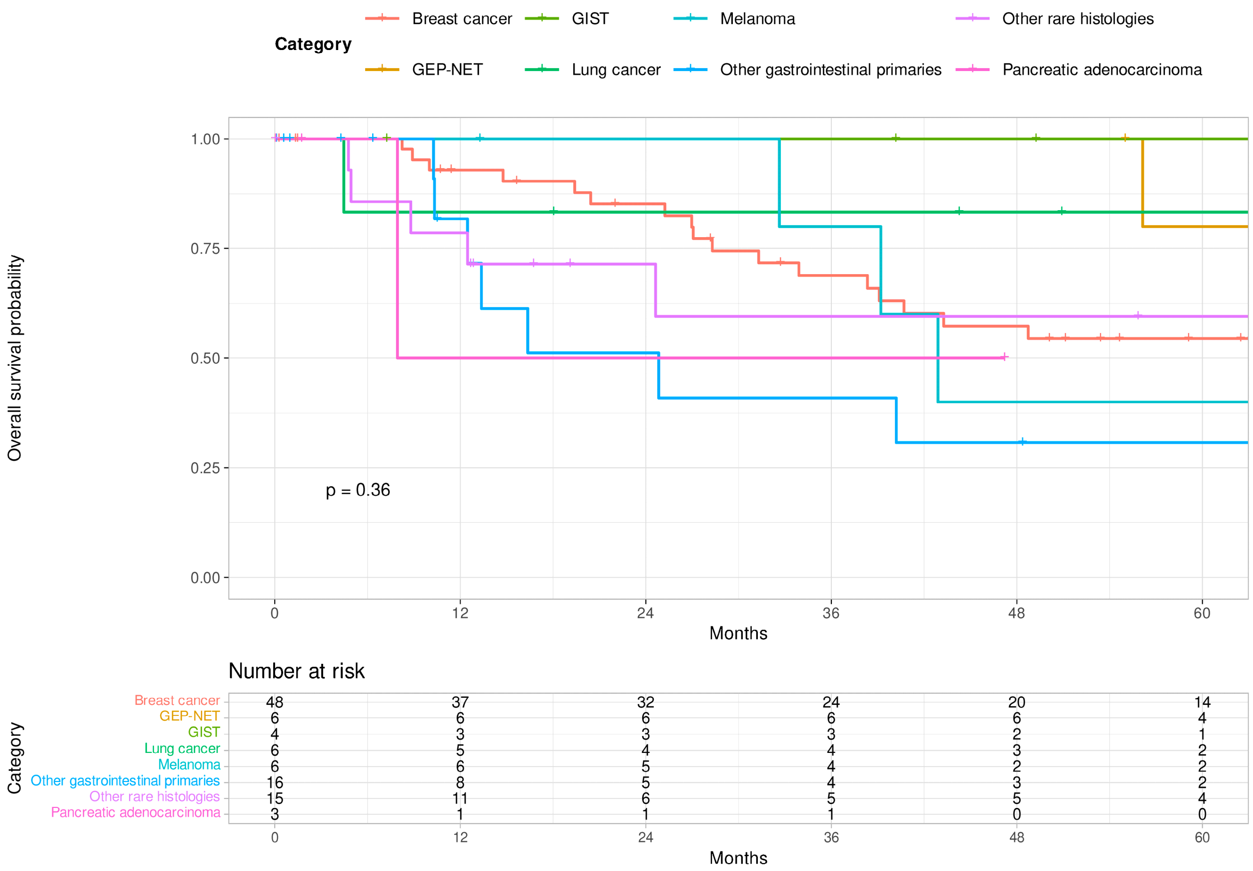

Figure S2. Kaplan–Meier survival curves of patients who were treated with radical intent upfront, stratified according to primary tumor histology.

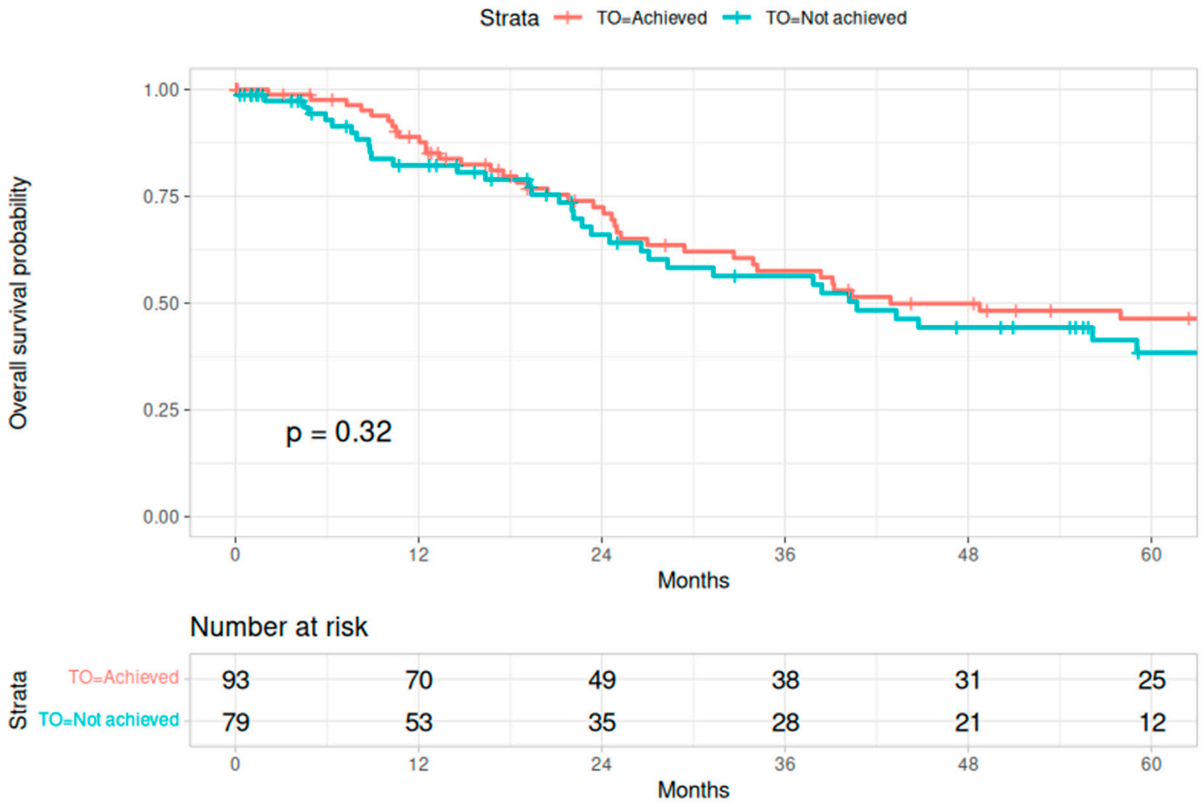

Figure S3. Kaplan–Meier survival curves of MWA procedures stratified according to textbook outcome (TO) achievement.

Table S3. Recurrence pattern and treatments

Variable	N = 112[1]
First recurrence site	
• Liver	101 (90%)
• Peritoneum	3 (2.7%)
• Bone	2 (1.8%)
• Brain	1 (0.9%)
• Breast	1 (0.9%)
• Chest wall	1 (0.9%)
• Psoas muscle	1 (0.9%)
• Liver and extrahepatic	1 (0.9%)
• Lymph node	1 (0.9%)
Second recurrence site	
• Lung	7 / 16 (44%)
• Bone	5 / 16 (31%)
• Lymph node	3 / 16 (19%)
• Peritoneum	1 / 16 (6.3%)
Third recurrence site	
• Liver	2 / 4 (50%)
• Brain	1 / 4 (25%)
• Lung	1 / 4 (25%)
Treatment of first recurrence	
• MWA	48 (43%)
• Chemotherapy	47 (42%)
• Resection	7 (6.3%)
• TACE	5 (4.5%)
• BSC	3 (2.7%)

[1] n / N (%).

MWA, microwave ablatio; TACE, transarterial chemoembolization; BSC, bast supportive care.

Table S4. Univariable and Multivariable analysis of factors associated with Overall Survival.

	Univariable			Multivariable		
**Variable**	**HR**	**95% CI**	***p***	**HR**	**95% CI**	***p***
Breast cancer	0.94	0.57, 1.55	0.8			
Lung cancer	1.00	0.36, 2.75	>0.9			
Melanoma	0.77	0.28, 2.13	0.6			
GEP-NET	0.33	0.08, 1.34	0.12			
Other gastrointestinal primaries	2.10	1.03, 4.29	0.042			
Other rare histologies	1.16	0.57, 2.35	0.7			
Pancreatic adenocarcinoma	3.62	1.30, 10.1	0.014			
Histological prognostic group*:						
• Favourable (ref.)	-	-	-	-	-	-
• Unfavourable	1.25	0.72, 2.16	0.4	1.37	0.74, 2.53	0.3
• Other	1.27	0.60, 2.69	0.5	1.16	0.49, 2.76	0.7
Age	1.01	0.99, 1.04	0.2			
Sex (male)	0.81	0.44, 1.49	0.5			
ECOG PS ≥ 2	4.43	1.37, 14.3	0.013	6.06	1.78, 20.7	0.004
Diabetes	1.34	0.54, 3.36	0.5			
Number of nodules:						
• 1 (ref.)	-	-	-			
• 2 -3	1.07	0.60, 1.88	0.8			
• > 3	1.47	0.73, 2.97	0.3			
Major diameter	1.08	0.95, 1.22	0.2			
TBS	1.07	1.00, 1.15	0.058	1.09	1.01, 1.18	0.032
Bilobar disease	1.55	0.84, 2.88	0.2			
Macrovascular tumor invasion	3.63	0.49, 26.7	0.2			
Extrahepatic metastasis	1.94	1.00, 3.78	0.050	1.29	0.40, 4.21	0.7
Previous Liver Resection	0.39	0.14, 1.09	0.073			
Previous Liver Ablation	1.09	0.52, 2.29	0.8			
Previous Liver Treatments	0.78	0.42, 1.45	0.4			
Number of previous liver surgery	0.81	0.51, 1.28	0.4			
Number of previous liver treatments	0.94	0.63, 1.39	0.7			
Surgical Technique:						
• Video-assisted approach (ref.)	-	-	-	-	-	-
• Percutaneous approach	2.08	1.25, 3.45	0.005	2.44	1.38, 4.31	0.002
Concurrent procedure	0.46	0.24, 0.89	0.021			
Surgical Intent:						
• Radical (ref.)	-	-	-			
• Non-radical	3.34	1.92, 5.82	<0.001			
Operative time (min)	0.99	0.99, 1.00	0.014			
Nodules treated	0.99	0.83, 1.18	0.9			
Duration, Σ (min)	0.99	0.95, 1.03	0.7			
Power (Watt)	0.98	0.96, 1.01	0.2			
LOS	0.84	0.71, 1.01	0.061			
Postoperative complications	0.44	0.16, 1.20	0.11			
Clavien-Dindo ≥ 3	4.09	1.29, 13.0	0.017			
CCI ≥ 26.2	0.97	0.13, 7.02	>0.9			
Readmission within 30 days	3.45	1.22, 9.76	0.019			
Recurrence	1.41	0.82, 2.41	0.2			
Persistence (No CR)	2.04	1.17, 3.55	0.012			
Textbook Outcome:						

• Achieved (ref.)	-	-	-
• Not achieved	1.30	0.78, 2.16	0.3
Efficacy (CR + No 90-day mortality)	0.45	0.26, 0.78	0.005

*Histological prognostic group. Favourable: breast, GIST, GEP-NET; Unfavourable: lung, melanoma, other gastrointestinal primaries, pancreatic adenocarcinoma; Other: Other rare histologies.

CI, Confidence Interval; HR, Hazard Ratio; GIST, gastrointestinal stromal tumour; GEP-NET, gastroenteropancreatic-neuroendocrin tumour; ECOG PS, Eastern Cooperative Oncology Group Performance Status; TBS, tumour burden score; LOS, length of hospital stay; CCI, comprehensive complication index; CR, complete response;
